# Deep Learning Techniques for the Effective Prediction of Alzheimer’s Disease: A Comprehensive Review

**DOI:** 10.3390/healthcare10101842

**Published:** 2022-09-23

**Authors:** K Aditya Shastry, V Vijayakumar, Manoj Kumar M V, Manjunatha B A, Chandrashekhar B N

**Affiliations:** 1 Department of Information Science and Engineering, Nitte Meenakshi Institute of Technology, Bangalore 560064, India; 2 School of Computer Science and Engineering, The University of New South Wales, Sydney, NSW 2052, Australia; 3 School of NUOVOS, Ajeenkya D Y Patil University, Pune 412105, India; 4 Swiss School of Business and Management, 1213 Geneva, Switzerland

**Keywords:** deep learning, health informatics, Alzheimer’s disease

## Abstract

“Alzheimer’s disease” (AD) is a neurodegenerative disorder in which the memory shrinks and neurons die. “Dementia” is described as a gradual decline in mental, psychological, and interpersonal qualities that hinders a person’s ability to function autonomously. AD is the most common degenerative brain disease. Among the first signs of AD are missing recent incidents or conversations. “Deep learning” (DL) is a type of “machine learning” (ML) that allows computers to learn by doing, much like people do. DL techniques can attain cutting-edge precision, beating individuals in certain cases. A large quantity of tagged information with multi-layered “neural network” architectures is used to perform analysis. Because significant advancements in computed tomography have resulted in sizable heterogeneous brain signals, the use of DL for the timely identification as well as automatic classification of AD has piqued attention lately. With these considerations in mind, this paper provides an in-depth examination of the various DL approaches and their implementations for the identification and diagnosis of AD. Diverse research challenges are also explored, as well as current methods in the field.

## 1. Introduction

DL has become a prominent issue in the ML domain in the past few years. ML can be utilized to tackle issues in different sectors. Neuroscience is included in this list. It is well known that detecting malignancies and functioning regions in cognitive systems has been a huge challenge for scientists over the years. The standard approach of detecting the variation in blood oxygen levels can be applied for this purpose. However, completing all the processes can take too long on certain occasions [1]. One benefit of DL approaches over typical ML methods is that the reliability of DL techniques grows with the phases of learning. The efficiency of DL methods tends to rise greatly as more information is provided to them, and they outperform conventional techniques [2]. This is similar to the human brain, which learns more as new information becomes available on a daily basis [2].

The domains of detecting AD have lately attracted a lot of research interest. AD is the most frequent type of dementia. Its symptoms can arise well after 60 years of age, and the chance of developing the illness increases with advancing age. AD can be split into seven phases. The first is the normal phase, which is accompanied by behavioral and mood variations, along with impaired functioning. The second phase is typical ageing amnesia, in which patients are unable to recollect names as easily as they could in the previous 5 to 10 years. “Mild cognitive impairment” (MCI) is the third phase, and patients’ frequent enquiries are a sign. Mild AD forms the fourth phase, and signs include a reduced capacity to handle finances and to make food for visitors. Stage 5 of AD is moderate AD, which manifests itself as a deficiency in fundamental regular tasks. The sixth phase of AD is intermediate-severe AD, which is marked by a loss of the capacity to perform everyday tasks. Extreme AD is the final phase, and an indication is that the person needs assistance in day-to-day tasks to survive [3].

As it can be observed, AD possesses seven phases, each of which has its own set of characteristics, so it is critical to know which phase of symptoms individuals are experiencing. Furthermore, AD therapies cover a diverse range of areas and benefits. The first is therapy that aids patients in maintaining their psychological health. A further benefit is that therapy aids in the management of behavioral issues. Third, therapy reduces or slows the progression of disease symptoms. As a result, the relevance of classification and prognosis can be seen, which is why researchers in this sector use DL methods [4].

The emphasis of this study is on the various DL methods as well as the various real-world applications of DL for AD detection. The subsequent sections of the paper are as follows: The transition from ML to DL approaches for AD prediction is discussed in the Section 2. The Section 3 discusses the various DL strategies for detecting AD. The Section 4 uses real-world case studies to show how DL can be used in the field of Alzheimer’s diagnosis. In Section 5, the various research problems encountered during AD prediction utilizing DL approaches are explored. Section 6 is devoted to the paper’s discussion. The paper’s conclusion and future scope are offered at the end.

## 2. Transformation from ML to DL Approaches for the Effective Prediction of AD

During the last decade, ML has been employed to discover neuroimaging indicators of AD. Several ML technologies are now being used to enhance the diagnosis and prognosis of AD [5]. The authors of [6] used a “support vector machine (SVM)” to accurately categorize steady MCI vs. progressing MCI in 35 occurrences of control subjects and 67 MCI instances. In most ML procedures for bio-image identification, slicing is prioritized, but recovery of robust shape features has mostly been ignored. In several circumstances, however, extracting convincing qualities from a feature space could eliminate the necessity for image classification [7]. Most early studies relied on traditional shape features such as “Gabor filters” and “Haralick texture” attributes [8,9]. DL is defined as a novel domain of ML research that was launched with the purpose of bringing ML nearer to its initial objective: “artificial intelligence (AI)”. To interpret textual, voice, and multimedia files, the DL architecture often requires more abstraction and representation levels [10].

The authors of [11] provide a comparative analysis of classical ML and DL techniques for the early diagnosis of AD and the development of mild cognitive impairment to Alzheimer’s disease. They examined sixteen techniques, four of which included both DL and ML, and twelve employed only DL. Using a combination of DL and ML, an accuracy rate of 96% was attained for feature selection and 84.2% for MCI-to-AD transformation. Utilizing CNN in the DL method, an attribute selection accuracy of 96.0% and a MCI-to-AD conversion predictive performance of 84.2% were obtained. In particular, the authors discovered that categorization ability could be enhanced by combining composite neuroimaging with serum biomarkers.

**Figure 1:**
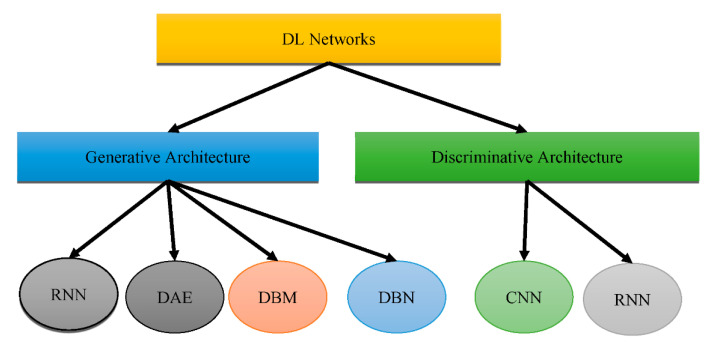
Types of DL architectures.

According to the study in [12], it is obvious that DL approaches for feature extraction and the ML strategy of classification using a SVM classifier are extremely effective for AD diagnosis and prediction. It has also been noted that prognosis and treatment based on many modalities fare better than those based on a single modality. Recent developments show a rise in the application of DL algorithms for the study of medical images, allowing for quicker interpretation and more improved precision than a human clinician. Figure 1 shows that DL could be placed into two groups: “generative architecture” and “discriminative architecture”.

**Figure 2:**
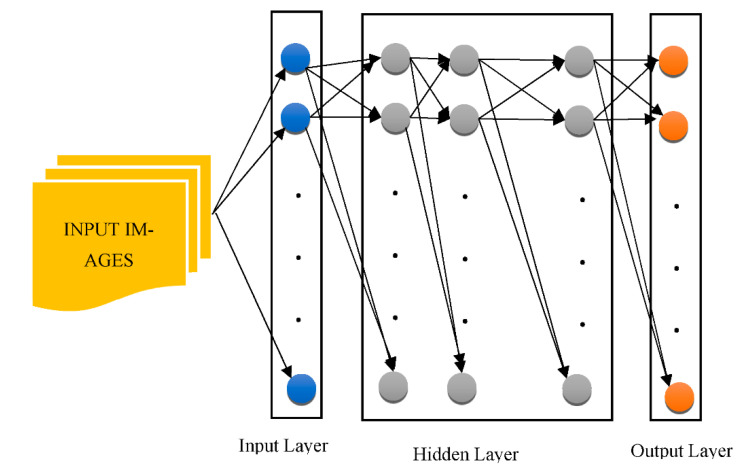
The architecture of a generalized CNN.

The “Recurrent Neural Network (RNN)”, “Deep Auto-Encoder (DAE)”, “Deep Boltzmann Machine (DBM)”, and “Deep Belief Networks (DBN)” are the four kinds of “generative architecture”, whereas the “Convolutional Neural Network (CNN)” and RNN are the two kinds of “discriminative architecture”. The structurally complex transformations and local derivative structures were recently discovered as current segmentation techniques for Phyto analytics by many scientists [11,12,13]. These descriptions are referred to as hand-crafted traits since they were created by people to extract characteristics from photos. A major aspect of employing these characteristics was to utilize vectors to locate a part of a picture, whereupon the created pattern is extracted. The SVM then receives the characteristics obtained by the customized approach [14] as a form of predictor. The best characteristics extract characteristics from a database. Several of the most widely used and concise descriptors rely on DL to achieve this [15,16]. As shown in Figure 2, the CNN is used to pull descriptions out of the images for this reason.

CNNs are particularly good at retrieving general features [17]. Various layers of approximations are formed when a deep network has been built on a large volume of imagery. The first-layer characteristics, for example, are like “Gabor filters” or color objects, which can be used for a wide range of picture issues and repositories [18]. “Deep neural networks (DNN)” can be employed on bio-image records; however, this method necessitates a large volume of information that is difficult to come by in many circumstances [19]. The information augmentation procedure is an answer to this situation, as it could customize the preliminary data using its own approach, allowing it to build information. Reflection, translation, and pivoting original imageries to generate opposing portrayals are certain popular information augmentation processes [20]. Customizing the picture’s luminosity, intensity, as well as brightness could also produce diverse images [21,22]. “Principal component analysis (PCA)” is another commonly utilized technique for information augmentation. Certain essential elements are inserted into a PCA once they have been scaled down to a smaller proportion [23,24]. The major goal of this procedure is to display only the picture’s highly appropriate features. “Generative adversarial networks” have been used in recent studies [25,26] to combine images that vary with the primary ones. This strategy necessitates the creation of a separate domain [27,28].

The images generated, however, are not reliant on modifications in the image database. As a result, different techniques may be applied depending upon the issue. For instance, element-wise computation was used to mimic random noise in radar altimeter imagery in [29]. Ductility was used in [30] to mimic the process of stretching in prostate chemotherapeutics. An alternative technique that takes advantage of DL is to adjust a pre-trained DL model, such as a CNN, on fresh data reflecting a different challenge. This method takes advantage of a pre-trained CNN’s shallow depth layers. Fine-tuning (also known as “tuning”) is a technique for stretching the learning phase on a new image dataset. This strategy significantly decreases the computing expenses of learning new information and is suited for modest populations. Another advantage of fine-tuning is that it enables scientists to readily study CNN combinations because of lower processing expenses. Such configurations could be created with multiple pre-trained CNNs and a variety of hyperparameters.

CNNs are also used as attribute extractors in certain investigations [31]. SVM with quadratic or regular kernels plus “logistic regression” and “extreme ML random forest” or “XGBoost” and “decision trees” are used for classifications [32]. Shmulev et al. [33] evaluated the findings acquired via the CNN technique to those obtained through alternative classifiers that only analyzed characteristics derived by CNN and determined that the latter works better than the former. Rather than being deployed explicitly for visual information, CNNs could be utilized on pre-extracted characteristics. This is particularly pertinent whenever a CNN is administered to the outcomes of different regression methods and whenever diagnostic ratings are matched across other model parameters and magnetic resonance characteristics.

CNNs could also be used to analyze non-Euclidean environments such as clinical charts or cerebral interface pictures. Morphological MRIs could be used with different designs. Various perceptron variants, such as a “probabilistic neural network” or a “stacked of FC layers,” were used in various studies. Several studies used both “supervised” (deep polynomial networks) and “unsupervised” (deep Boltzmann machine and AE) designs to retrieve enhanced interpretations of attributes, whereas SVMs are primarily used for classification [34]. Imagery parameters such as texturing, forms, trabecular bone, and environment factors are subjected to considerable pre-processing, which is common in non-CNN designs. Furthermore, to further minimize the dimensions, the integration or extraction of attributes is commonly utilized. On the other hand, DL-based categorization techniques are really not limited to cross-sectional structural MRIs. Observational research could combine data from various time frames while researching relatively similar topics.

In [35], the authors developed an SVM with kernels that permitted antipsychotic MCI to be switched to AD while the other premonitory categories of AD were removed. They were able to achieve a 90.5 percent cross-validation effectiveness in both the AD and NC studies. They were also 72.3 percent accurate in predicting the progression of MCI to AD. Regarding the extraction of attributes, two methods were utilized:

- “Free Surfer” is an application for cerebral localization with cortex-associated information.
- The “SPM5 (Statistical Parametric Mapping Tool)” is a device for the mapping of statistical parameters.

Researchers further found that characteristics ranging from 24 to 26 are the most accurate predictors of MCI advancing to AD. They also discovered that the width of the bilateral neocortex may be the most important indicator, followed by right hippocampus thickness and APOE E”4 state. Costafreda et al. [36] employed hippocampus size to identify MCI patients who were inclined to progress to AD. A number of 103 MCI patients from “AddNeuroMed” were used in their research. They employed the “FreeSurfer” for information pre-processing and SVM with a semi-Stochastic radial basis kernel for information categorization. Following model training on the entire AD and NC datasets, researchers put it into practice. In less than a year, they were able to achieve an accuracy of 85 percent for AD and 80 percent for NC. They concluded that hippocampus alterations could enhance predictive efficacy by consolidating forebrain degeneration.

According to a comprehensive analysis of various SVM-centered studies [37], SVM is a commonly used technique to differentiate between AD patients and apparently healthy patients, as well as between steady and progressing subtypes of MCI. Regarding diagnoses, advancement projections, and therapy outcomes, functional and structural neuroimaging approaches were applied. Eskildsen et al. [38] found five important ways to tell the difference between stable MCI and MCI that is becoming worse.

To differentiate and diagnose AD, the researchers in [39] studied 135+ AD subjects, 220+ CN patients, and 350+ MCI patients. They trained on the neuroimaging utilizing information from ADNI. To differentiate AD patients from CN patients, they employed “neural networks” and “logistic regression”. The metrics were determined to have extensive brain properties. Rather than relying on specific parts of the brain, important properties such as volume and thickness were determined.

Because of its capacity to gradually analyze multiple levels and properties of MRI and PET brain pictures, the authors of [40] advised using cascading CNNs in 2018. Since no picture segmentation was used in the pre-treatment of the information, no skill was necessary. This trait is widely seen as a benefit of this technique over others. The attributes were extracted and afterwards adapted to the framework in the other techniques. Depending on the ADNI dataset, their research included 90 plus NC and AD subjects, with 200 plus MCI cases. The efficiency rate was greater than 90%.

The work in [41] suggested a knowledge-picture recovery system that is based on “3D Capsules Networks (CapsNets)”, a “3D CNN”, and pre-treated 3D auto-encoder technologies to identify AD in its early phases. According to the authors, 3D CapsNets are capable of quick scanning.

Unlike deep CNN, however, this strategy could only increase identification. The authors were able to distinguish AD with a 98.42% accuracy. The authors of [42] looked at 407 normal participants, 418 AD patients, 280 progressing MCI patients, and 533 steady MCI instances from an institution. They practiced on 3D T1-weighted pictures using CNNs. The repository they used was ADNI. They looked at CNN operations to identify AD, progressing MCI, and stable MCI. Whenever CNNs were utilized to separate the progressing MCI individuals from the steady MCI patients, there was a 75% accuracy rate. The researchers in [43] developed an algorithm that used MRI scans to determine medical symptoms. The maximum number of cases that researchers could use was 2000 or more, and they chose to work on the ADNI repository.

“DSA-3DCNN” was reported to be quite accurate compared to alternative contemporary classifiers in diagnosing AD that relied on MRI scans by Hosseini-Asl et al. [44]. The authors demonstrated that distinguishing between AD, MCI, and NC situations can improve the retrieval of characteristics in 3D-CNN. With respect to analysis, the cerebral extraction technique used seven parameters. The FMRIB application package was utilized. This collection offers technologies to help MRI, fMRI, and DTI neuroimaging information, in addition to outlining the method of processing the information. By eliminating quasi-cerebral tissues from head MRIs, PET was utilized to categorize them into cerebral and non-cerebral imageries (a vital aspect of any assessment). In BET, no prior treatment was required, and the procedure was quick.

## 3. Diagnosis and Prognosis of AD Using DL Methods

DL is a subfield of ML [45] that discovers characteristics across a layered training process [46]. DL approaches for prediction and classification are being used in a variety of disciplines, such as object recognition [47,48,49] and computational linguistics [50,51], which together show significant improvements over past methods [52,53,54]. Since DL approaches have been widely examined in the past few years [55,56,57], this section concentrates on the fundamental ideas of “Artificial Neural Networks (ANNs)”, which underpin DL [58]. The DL architectural schemes used for AD classification and prognosis assessment are also discussed. NN is a network of connected processing elements that have been modeled and established using the “Perceptron”, the “Group Method of Data Handling” (GMDH), and the “Neocognitron” concepts. Because the single layer perceptron could only generate linearly separable sequences, these significant works investigated effective error functions and gradient computational algorithms. Furthermore, the back-propagation approach, which utilizes gradient descent to minimize the error function, was implemented [59].

After detection, a person with AD can expect to live for an average of 3 to 11 years. Certain individuals, nevertheless, may survive for 20 years or more after receiving a diagnosis. The prognosis typically relies on the patient’s age and how far the illness has advanced prior to detection. The sixth most frequent cause of mortality in the US is AD. Other ailments brought on by the problems of AD can be fatal. For instance, if a person with AD has trouble swallowing, they may suffer from dehydration, malnourishment, or respiratory infections if foods or fluids enter their lungs. The individuals responsible for the patient’s care are also directly and significantly impacted by AD in addition to the patients themselves. Caregiver stress condition refers to a deterioration in the psychological and/or physical well-being of the individual caring for the Alzheimer’s sufferer and is another persistent complication of AD in this regard.

Rapid progress in neuroimaging techniques has rendered the integration of massively high-dimensional, heterogeneous neuroimaging data essential. Consequently, there has been great interest in computer-aided ML techniques for the integrative analysis of neuroimaging data. The use of popular ML methods such as the Support Vector Machine (SVM), Linear Discriminant Analysis (LDA), and Decision Trees (DT), among others, promises early recognition and progressive forecasting of AD. Nevertheless, proper pre-processing processes are required prior to employing these methods. In addition, for classification and prediction, these steps involve attribute mining, attribute selection, dimensionality reduction, and feature-based classification. These methods require specialized knowledge as well as multiple time-consuming optimization phases [5]. Deep learning (DL), an emerging branch of machine learning research that uses raw neuroimaging data to build features through “on-the-fly” learning, is gaining significant interest in the field of large-scale, high-dimensional neuroimaging analysis as a means of overcoming these obstacles [59].

### 3.1. Gradient Computation

**Figure 3:**
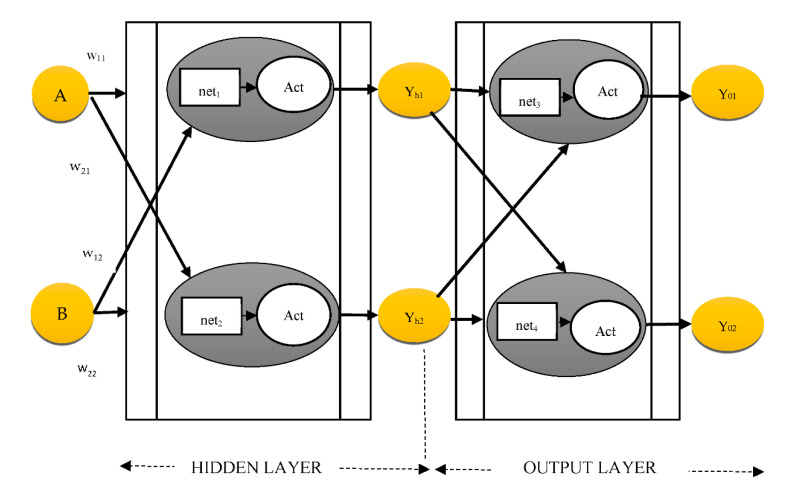
MLP procedure (where Act signifies activation function, w represents weights, and Net denotes network).

The error between the training algorithm output and the intended result is calculated using the back-propagation process. The back propagation formula computes the difference several times, altering the weights each time and halting until the difference is no longer adjusted [60]. The technique of creating an ANN using a “multi-layer perceptron” is depicted in Figure 3.

The weights are changed using a “back-propagation” process until the differential value reaches 0, once the first erroneous value is obtained using the least squares approach from the hypothetical random distribution weight. The network weights are adjusted until the divergence score reaches 0, after the preliminary error score is determined from that of the hypothetical random distribution weight using the least squares approach. For instance, Equation (1) updates the *w*_21_ of Figure 3:

(1) $$w_{21}\left(m+1\right)=w_{21}m-\frac{\partial ErrY_{out}}{\partial w_{21}}$$

(2) $$ErrY_{out}=\frac{1}{2}{\left(y_{t1}-y_{01}\right)}^{2}+\frac{1}{2}{\left(y_{t2}-y_{o2}\right)}^{2}$$

The *ErrY*_out_, which is the sum of error *y*_01_, is shown in Equation (2). The parameters *y*_o2_., *y_t_*_1_, and *y_t_*_2_ are obtained from the supplied information. The chain rule could be used to determine *ErrY*_out_’s partial derivative regarding *w*_21_:

(3) $$\frac{\partial ErrY_{out}}{\partial w_{21}}=\frac{\partial ErrY_{out}}{\partial y_{o1}}.\frac{\partial y_{o1}}{\partial net1}.\frac{\partial net1}{\partial w_{21}}$$

Similarly, the chain rule updates *w*_11_ in the hidden layer, as indicated in Equation (4):

(4) $$\frac{\partial ErrY_{out}}{\partial w_{11}}=\frac{\partial ErrY_{out}}{\partial y_{h1}}\times \frac{\partial y_{h1}}{\partial net_{1}y}\times \frac{\partial net_{1}}{\partial w_{11}}$$

### 3.2. DNNs in the Real World

Because backpropagation utilizes a “gradient descent” approach to determine the weights of every layer, and since it is piled downwards from the output nodes, a diminishing gradient phenomenon develops, in which the divergence number reaches 0 prior to finding the optimal value. Whenever the sigmoid is differentiated, the peak value is 0.25, and as it multiplies, it draws nearer to 0. This is known as the diminishing gradient phenomenon, and it is a key stumbling block for DNNs. The problem of the diminishing gradient has been extensively studied [61]. One of the results of this endeavor was the replacement of the sigmoid function, which is an activation function, with several different measures, including the “hyperbolic tangent function”, “ReLu”, and “Softplus” [62,63]. The “hyperbolic tangent function” extends the sigmoid’s spectrum of derivative scores. The most commonly utilized activation function is the “ReLu”, which substitutes a number with 0 when it becomes 0 and utilizes the number when it becomes greater than 0. It will become plausible to alter the weights from vanishing down to the very first layer via layered hidden units as the derivatives approaches 1 whenever the value is greater than 0. This basic strategy provides an easy implementation of DL by allowing numerous levels to be built. When ReLu reaches zero, the “Softplus” method is substituted, which uses a gentle fall mechanism.

While weights are calculated accurately using the gradient descent approach, it normally consumes a lot of time to compute, since all the information must be distinguished at every iteration. To address performance and reliability difficulties, improved gradient descent algorithms were devised in conjunction with the activation function. The “Stochastic Gradient Descent (SGD)”, for instance, employs a portion of the complete information, which is selected randomly for quicker and much more regular iterations [64], and it has been expanded to “Momentum SGD” [65]. The “Adaptive Moment Estimation” (Adam) is presently among the most common gradient descent algorithms.

### 3.3. DNN Architectures

**Figure 4:**
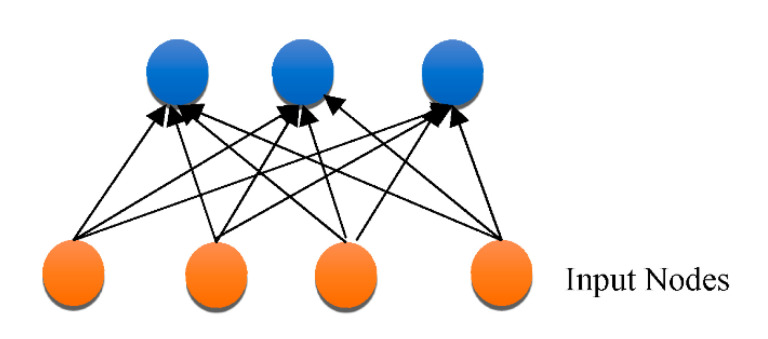
Architecture of RBM.

**Figure 5:**
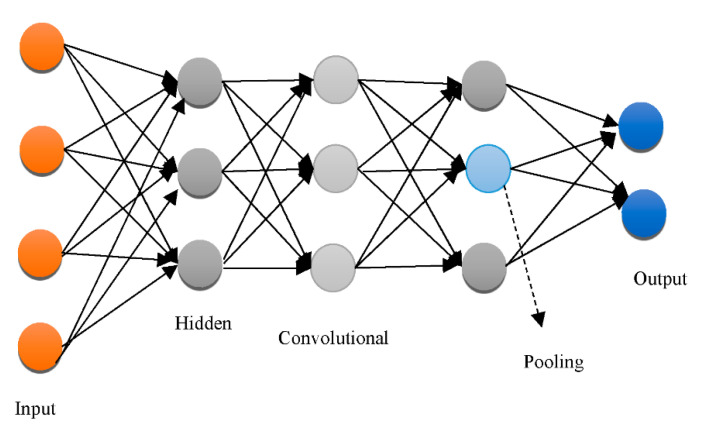
CNN architecture.

**Figure 6:**
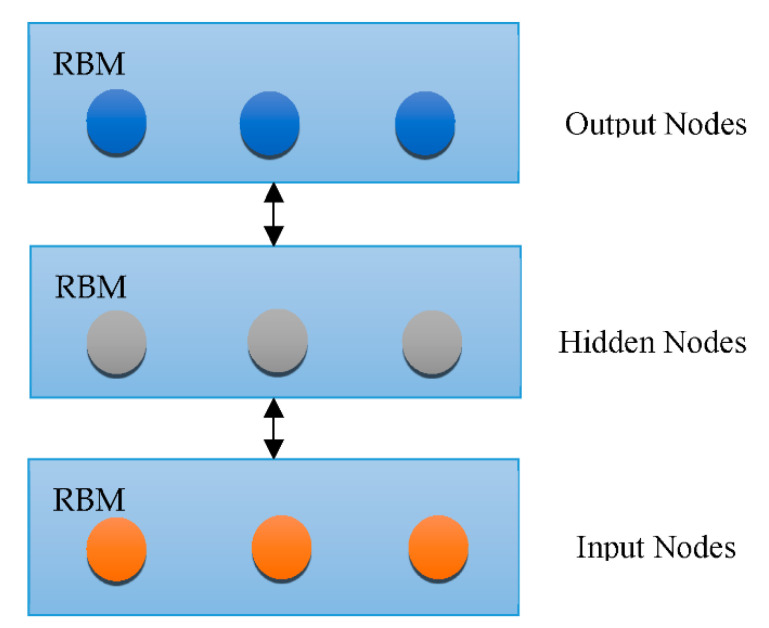
RBM architecture.

**Figure 7:**
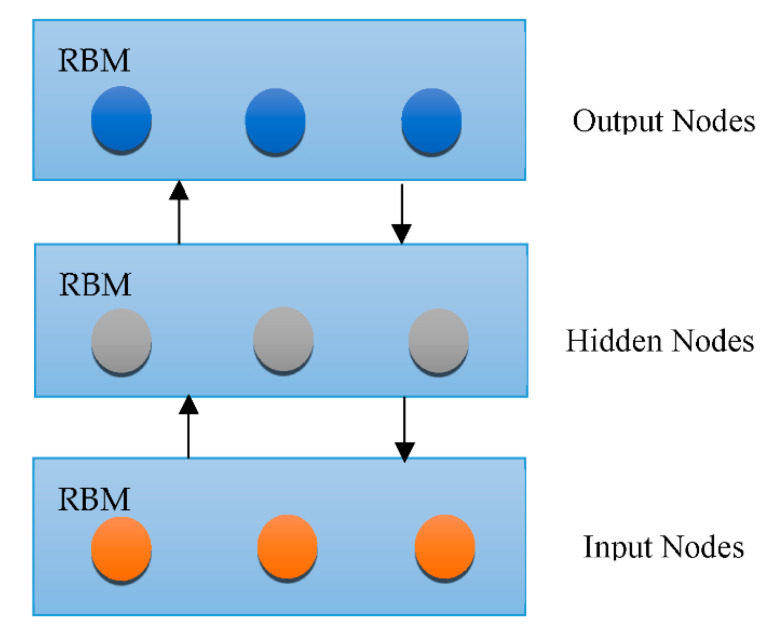
DBN architecture.

**Figure 8:**
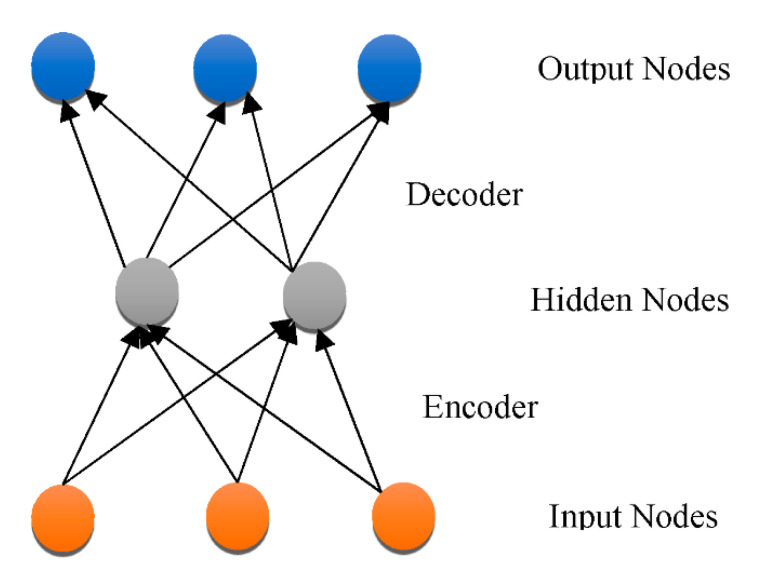
AE architecture.

**Figure 9:**
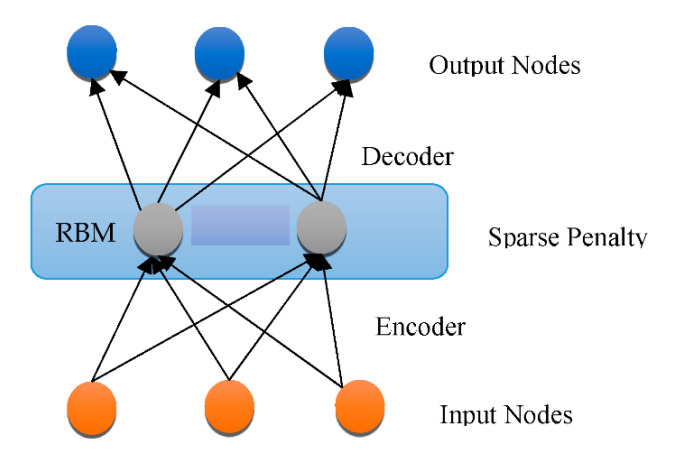
Sparse AE architecture.

**Figure 10:**
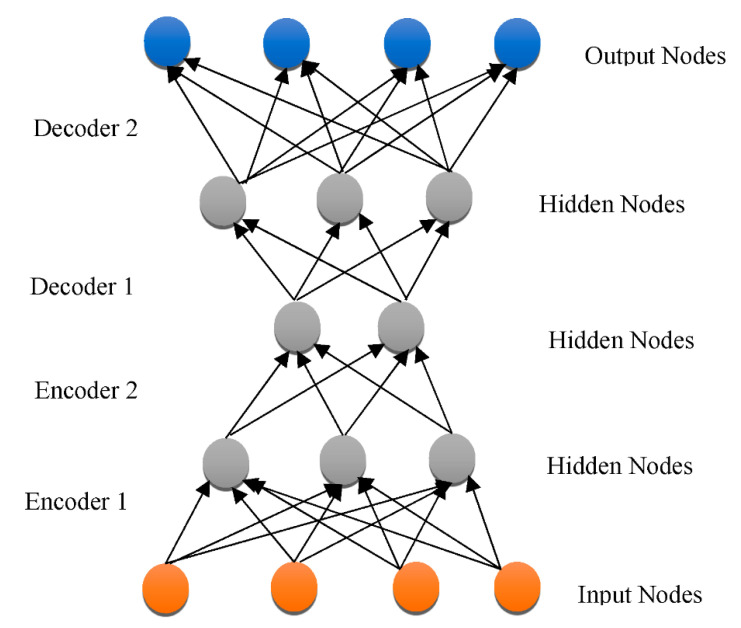
Stacked AE architecture.

Overfitting has also contributed immensely to the development of DL [66], with attempts to handle the issue at an individual and collective scale. One of the earliest models created to tackle the generalization error was the “Restricted Boltzmann Machine (RBM)” [58]. It combines the RBMs evolved in the “Deep Boltzmann Machine (DBM)”, which is a denser architecture [67]. The “Deep Belief Network (DBN)” is a supervised learning system that extracts information out of each tier level to link unstructured variables. DBN outperformed conventional algorithms, which is one of the reasons that DL has become so prominent. Although DBN eliminates the possibility of hyperparameters by employing RBM to minimize weight initialization, CNN effectively limits the number of hyperparameters by integrating convolution and pooling levels, resulting in a decrease in difficulty. Due to its sufficiency, CNN is frequently utilized in the domain of visual recognition. “RBM, DBM, DBN, CNN, Auto-Encoders (AE), sparse AE, and stacked AE” are all depicted in Figure 4, Figure 5, Figure 6, Figure 7, Figure 8, Figure 9 and Figure 10 respectively.

“Auto-encoders (AE)” represent an unsupervised classification methodology that utilizes a back-propagation algorithm and SGD to allow the resulting value to approximate the data input. Owing to the diminishing gradients problem, AE activates dimensionality minimization, although it is hard to train. Sparse AE solves this problem by permitting just a minimal number of hidden layer units to be used [68]. “DBN, DNN, RBM, DBM, DBN, AE, Sparse AE, and Stacked AE” are DL algorithms that have been employed for AD diagnostic categorization up to this point. Every method was created to distinguish “cognitively normal controls (CN)” from “mild cognitive impairment (MCI)”, which is the premonitory phase of AD. Employing multi-modal neuroimaging information, every technique is utilized to forecast the transition of MCI to AD.

### 3.4. DL for Selection of Attributes from Neuroimaging Information

Structure and genomic indicators for AD have been identified using heterogeneous neuroimaging datasets. Pre-selected AD-specific areas, such as the hippocampal and neocortex, have been found to be critical markers for improving ML classification performance. DL algorithms were applied to identify characteristics from neuroimaging repositories.

In [69], the authors classified AD/CN with more than 86% efficiency using “stacked sparse autoencoders (SAEs)” and a “softmax” regression layer. To retrieve additional data from multichannel brain images [70,71,72], they utilized SAE and a “SoftMax logistic regressor”, along with a zero-mask tactic for information fusion, in which one of the therapies is arbitrarily concealed by substituting the input parameters with 0 to converge distinct kinds of information for SAE. The DL method improved AD/CN classification performance by 90%. The authors of [73] achieved more than 84 percent AD/CN prediction performance and an 82 percent MCI transition accuracy rate using SAE for pre-training and DNN for the final phase. A CNN that has demonstrated exceptional results in the domain of machine vision was also used to diagnose AD using heterogeneous neuroscience datasets.

The authors in [74] employed feature maps to convert localized imageries into elevated attributes from raw MRI imageries for the “3D-CNN”, resulting in an 87-plus percent accuracy for AD/CN categorization. They raised the efficiency to more than 89% by testing two “3D-CNNs” on distinct neuroimage regions collected from “MRI” and “PET” data, subsequently merging the findings to execute a “2D CNN” [74]. In [75,76], the authors demonstrated more than 79% accuracy for AD/CN identification using two alternative 3D CNN algorithms (basic “VoxCNN” and “residual neural networks (ResNet)”). This was the first study showing that the subjective segmentation process was redundant. In [77,78], the authors took 2D segments of the hippocampus area in the radial, longitudinal, and frontal planes and used “2D CNN” to classify AD/CN with an 85-plus percent accuracy.

In [78], the researchers used an information learning strategy to pick configural regions from MR images built on AD-linked structural signs and then applied “3D CNN” on these. This method employed three separate sets of information (ADNI-1 for learning, ADNI-2 for assessment, and MIRIAD for validating) to produce sufficiently better accuracies of more than 91 percent for AD/CN diagnosis from “ADNI-2” and “MIRIAD”, correspondingly, and 75-plus percent for MCI transformation prognosis on “ADNI-2”. The work [79] employed three-dimensional CNN architectures to capture the quasi-association across MRI and PET patterns on participants for both MRI and PET scans and utilized the learnt network to infer PET characteristics for patients with only MRI information. The AD/CN classification performance in this research was more than 92% accurate, and the MCI transformation accuracy rate was more than 72%.

In [80], the researchers used SAE with “3D CNN” on MRI and FDG PET scans to achieve a 90% accuracy in AD/CN categorization. In [81], the scientists adopted a blend of 2D CNNs and RNNs to generate intra-slice and cross-attributes after decomposing 3D PET data into a series of 2D slices. The method identified AD/CN with 91 percent accuracy. When information is unbalanced, the risk of misinterpretation rises, and susceptibility falls. There have been 76 cMCI and 128 ncMCI individuals [82], and the observed sensitivity was less than 50%, which was poor. The work in [78] used 38 cMCI and 239 ncMCI patients and found that their sensitivity was less than 44%. The authors in [83] earlier revealed the first application of 3D CNN designs to heterogeneous PET scans, achieving more than 95% accuracy for AD/CN categorization and 84-plus percent accuracy for MCI-to-AD transition prognosis.

Table 1 shows the DL techniques for feature selection on neuroimaging data.

### 3.5. DL for Selection of Heterogeneous Neuroimaging Data

Heterogeneous neuroimaging information such as that from MRI and PET has indeed been frequently employed during DL to boost the effectiveness of AD/CN categorization and the prognostication of MCI-to-AD transformation: magnetic resonance for central nervous system functional degeneration, aβ peptide PET for frontal cortex oligomers accrual, and FDG-PET for glucose uptake biotransformation are examples. Thirteen studies used MRI scans, ten used FDGPET scans, twelve used both MRI and FDG-PET diagnostic tests, and one used both amyloid PET and FDG-PET scans. In comparison to MRI, PET scans performed significantly better in AD/CN diagnosis and/or detection of MCI-to-AD transition. The accuracy of two or more multimodal neuroscience types of information was better than that of a solitary neuroscience method.

To obtain the appropriate levels of performance accuracy, DL systems necessitate a huge amount of information. Due to the limited availability of neuroscience information, hybrid techniques that integrate classic ML methods for diagnosis categorization alongside DL techniques for attribute mining performed better and could be a useful alternative for dealing with such information. An “autoencoder (AE)” was used to interpret the original picture parameters, rendering them identical to the actual picture, while it was being used as input, allowing the restricted neuroscience information to be efficiently utilised. Though hybrid strategies have produced promising outcomes, they do not fully exploit DL, which pulls patterns from enormous volumes of neuroimaging information efficiently. The CNN, which specializes in retrieving properties from imagery, is the most widely utilized DL technique in machine vision research. Recently, 3D CNN techniques based on heterogeneous PET scans have performed effectively for AD/CN categorization and MCI-to-AD transition predictions.

## 4. Case Studies on the Diagnosis of AD Using DL and Related Technologies

Computer vision research forms an essential part of identifying and treating a variety of disorders. These kinds of images form a valuable resource for extracting diagnostics [84]. These are key aspects of the “Electronic Health Records (EHR)” and are typically analyzed by a group of specialists (“radiologists”). There are numerous picture types available, with MRI and PET being the most prevalent in AD. The use of AI to automate the analysis of these types of photographs has grown in popularity across time. In reality, AI is projected to play a major role in EHR research and not just for plain images:

- During the last 15 years, the utilization of such techniques and AI technologies in medical applications has skyrocketed. There seem to be three important aspects to consider, e.g., information quantity and quality have both improved. In this sense, the discipline is approaching Big Data.
- Efforts are being made to minimize human discrepancies, because radiologists are constrained by a variety of parameters, such as time or expertise, and are also likely to make errors [85,86].
- The emergence of AI in general and DL in particular is apparent. Adopting these systems for clinical use might not have been considered conceivable if they had not demonstrated such significant improvement during the past few decades [87,88].

Despite numerous articles published based on straightforward ML techniques such as SVM [86] and statistical techniques such as “independent component analysis (ICA)” [89], DL and CNN have captured healthcare imaging techniques in the last 5–10 years [90,91]. Such methods were also applied in a variety of computer-aided diagnostic scenarios. These could be broken down into the following groups:

- In detection with the help of computers (CADe), certain components in the imagery, such as structures or neurons, can be identified. CADe can also be used to identify areas of focus for scientists, like malignancies.
- Segmentation is the separation of complete picture portions from the rest of the imaging.
- Computer-aided Diagnosis (CADx) denotes a diagnostic based on particular data that can be described as a categorization task in plain terms. Medical photos are employed in this scenario, which emphasizes the necessity of CNN. In the context of AD, there are three classifications: NC, MCI, and AD.

The work in [91] mentions another intriguing area, which can be referred to as deep feature learning. It is focused on the creation and development of a plan that can retrieve important knowledge from data. It enables the acquisition of higher-level characteristics that are unseen to the naked eye and can be reused in a variety of contexts. It is frequently employed in Alzheimer’s. CADx is a preliminary step for retrieving valuable features from pictures or pre-training deep networks using diverse methods such as auto-encoders [92,93,94]. Nevertheless, this method is becoming obsolete, as it necessitates additional development steps and, thus, no substantial improvement in the overall performance is seen [95]. Deep feature extraction has not really addressed during the explanatory stage for these considerations. Finally, the ANN is the foundation for most experiments performed in the last 5–10 years. ANNs are typically taught under supervision, but their non-supervised applications are equally vital. In any case, imagery must be pre-processed for models to fully utilize it.

There are a few medical diagnostic pre-treatment approaches that appear in a broad range of articles connected to the AD automatic detection study. Initially, authors relied on individually created characteristics that necessitated the employment of quite sophisticated pre-treatment methods. The usage of CNN and auto-encoders for automatic attribute mining, on the other hand, makes the task relatively easy. Finally, two crucial operations must be emphasized: MRI image capturing and cranium removal.

Image registration is the process of matching a particular image to a source image, known as an overlay, to ensure that the same parts, including both images, reflect similar anatomical features [96,97]. Because similar data reside in almost all the imagery, it is easier for a CNN or an auto-encoder to identify a specific section of the imagery as important. There are numerous assessment methods [97] that can be used not only in neuroscience [94,98,99,100], but also in other medical domains such as melanoma [101].

Cranium peeling is the process of removing data from the cranium that is shown on MRI pictures, as the name suggests. The goal is to create an output photo that is as concise as possible, with only the data necessary for the assigned task. Clearly, neither of the most important indicators for AD can be detected in the cranium. As a result, other researchers [98,99,100] employ various strategies to remove the skull and other non-brain regions, or they use an imaging information source that already has the cranium removed [101]. It is important to note that endocrinal data are not discussed in research that employs PET imaging because these images do not contain quite so much extraneous information.

Additional universal methods, such as picture normalization, are available in addition to the abovementioned processes. Brightness normalization and geographical normalizing are two distinct examples. The first of these, known as whitening [102], is focused on modifying the spectrum of image pixels to include a specific criterion, such as lowering the pixels to a narrow timeframe or removing the average and subtracting the mean deviations. The next method entails resizing the pixels (or spatial information in three-dimensional images) to reflect a dedicated area (or capacity) [102]. For instance, every region of interest in [103] occupies 2 mm^3^ of volume. Face recognition could be thought of as a kind of geographic normalization in this context [102].

CNN has become increasingly significant in the diagnosis of AD in recent times. This is not to say that these algorithms have not been utilized before, but they were usually accompanied by other DL methodologies, such as the Shallow Extraction of features. CNN has been directly employed for the past 3 years, resulting in an effective model that is also far less time-consuming than initial efforts.

AlexNet was a watershed moment in DL history. It demonstrated how a CNN might achieve good picture prediction performance. Its principles were further explored in the decades to come, resulting in major designs, notably VGGNet, Inception, and ResNet. Even though CNNs were created to operate on ImageNet information, their exceptional outcomes have rendered them the preferred option for a wide variety of uses. Many DL frameworks already have these models built with ImageNet, so programmers can change them to fit their own needs.

It has not been any different when it comes to computer-aided diagnosis. Even though there are significant variations between the two zones that limit the effectiveness that could be achieved by adjusting these nets, this strategy has proven to be the most promising in practice not just with AD, but with other disorders such as vision loss, melanoma, as well as cervical cancer. Researchers have customized the “Inception V3” technique. The scientists of [104] experimented using both the “Inception V3” technique and a “ResNet50” model. An “Inception V3 model” was also utilized in [105] to classify 750-plus diverse illness types.

In reality, “Inception V3” and ImageNet are the most commonly used designs in the research works. Table 2 is a summary of the articles that use CNN to diagnose AD and the above disorders.

“LeNet5” is an older design that was inspired by [17]. “VoxCNN” is a “VGGNet”-derived cubic CNN. The “volumetric residual network VoxResNet” is a volumetric residual network. The first effort to explicitly build CNNs for AD diagnosis did not employ ImageNet parameters or parameter tuning; however, it did utilize two of these popular designs (“Inception V1”) and an earlier one from well before “AlexNet” [19]. The results recommend that utilizing more sophisticated and complicated designs might result in overfitting because of the insufficient information available. The authors integrated the two concepts and created a decision-making system that was almost 100% accurate.

Following this, another article [76] attempted to show that a CNN could simply be used to produce characteristics and categorize data in a totally automated fashion. The authors achieved this by making model creation as simple as possible, utilizing only 231 photos. They created a 3D CNN dubbed VoxCNN that was influenced by VGGNet and contrasted this to the VoxResNet framework. When compared to [44], the outcomes remained substantially inferior, although they did demonstrate the ease with which this technique might be implemented. Lastly, utilizing just the “Keras” toolkit to create the structures and “SciPy” to improve the image quality of pictures, the most contemporary article customized the “InceptionV3” model with 18F-FDG PET scans. The authors established that their algorithm quantitatively surpassed clinicians’ efforts considerably, particularly in predicting the development of AD more than 6 years in advance.

## 5. Research Challenges in DL for AD

AI in clinical applications faces several obstacles, some of which are comparable to those encountered by equivalent systems in other domains. The bulk of the issues are information-linked, though there are humanistic aspects to consider.

The first and most obvious difficulty is the scarcity of labeled information. Even though the severity of symptoms has decreased with the passage of time, they remain a major cause of worry for scholars, particularly when compared to other databases such as ImageNet. Although this central database has many pictures, OASIS Neurons currently provide MRI and PET information for 1098 people [107]. Although it is accurate to say that the number of trainings in therapeutic diagnostics is typically lower (ImageNet has 1000), this is not always the case.

Overfitting is frequently the result of this issue. The retrieval of numerous arbitrary regions from photographs, including two-dimensional and three-dimensional ones, was a frequent solution. This method is similar to how physicians examine images by areas [34]. Several methods extract the patches in a less random way, trying to use metadata to link many patches from each picture [108].

Information augmentation, which is less prevalent, or even the production of synthetic images, are further options. However, “transfer learning” appears to be the most popular strategy in recent articles. It is believed that while fine-tuning an Inception net or a ResNet, minimal training information will be required.

The unbalanced information problem is linked to the data availability issue. When compared to the positive class, the negative class is frequently found to be more prevalent. This is to be anticipated, given how much simpler it is to collect knowledge from normal subjects. To make matters harder, the negative group is frequently positively associated, and the positive class has a huge variety. According to studies on the matter, under-sampling the over-represented group is not a smart option, whereas over-sampling the under-represented category may be beneficial in certain situations [109].

Another major aspect is the architectural variation of the images. Apart from the various imaging types (MRI, fMRI, PET, and so on), these variations could be used in a variety of ways. The primary issue is whether to use the 3D information directly, as these images are usually in three dimensions, or to translate the images into two dimensions. Because it minimizes data redundancy, 3D information ought to be the default option [43]. Nevertheless, there has been a trend to convert the photos to 2D, since this is considerably faster and more efficient in preventing overfitting [103]. The mining 2D and 3D patches from images was compared in [43], and the results showed that the differences were not very extreme.

In addition to specific difficulties, there are various moral and philosophical issues at stake. HER are important pieces of information that not only restrict the quantity of photographs that may be collected, but also force one to use them very carefully. Confidence in AI is a related issue, as there is still a lot of misunderstanding regarding what AI is and how it works among the general population. AI is a hotly debated and ongoing area of study that is not limited to clinical uses [110]. The major issue usually involves the “black box” phenomenon. In Alzheimer’s-related literature [103], there have been some attempts to help with this problem, but this is not the norm.

DL also faces the challenges of transparency and reproducibility. The purpose of transparent DL is to enable the adequate explanation and communication of the results of a DL model. Even when all the parameters are known, it is difficult to grasp how the DL network operates, because its performance depends on the intricate relationships between many variables. The problem is coming up with solutions that make sense. Likewise, reproducing the code developed by one researcher is also a major challenge faced by DL. If more researchers are unable to replicate an experiment and obtain the same results as the original researchers, the hypothesis is invalidated. Therefore, failure to duplicate results diminishes the credibility of science.

## 6. Discussion

Alzheimer’s disease (AD) requires an accurate and timely diagnosis in order to begin successful therapy. Early detection of AD is very important for drug applications and, eventually, for diagnostic and therapeutic purposes. In this article, a comprehensive assessment was conducted for DL algorithms for the clinical categorization of AD based on brain signals. DL techniques have achieved accuracy levels of up to 95% in AD diagnosis and 84% in MCI transition prognosis. Even though it raises concern when investigations achieve high reliability with a small amount of information, particularly if the technique is susceptible to overfitting, the SAE process had a higher precision rate of 97-plus percent, while the amyloidosis PET scan had the lowest accuracy of 96.8%. When 3DCNN was performed on MRI data in addition to the attribute mining stage, the maximum accuracy for AD diagnosis remained greater than 86% [70]. Because of this, it has been shown that two or more different types of neuroscience data are more accurate than a single type of neuroscience data [52]. In classic ML, performance is influenced, including improved characteristics. Nevertheless, the more complicated the information, the more challenging it is to choose the best attributes. DL involves extracting the best information. DL is rapidly being employed for computer-aided diagnosis owing to its simplicity of usage and superior results. Since 2015, the volume of AD researchers utilizing a CNN that demonstrated higher image recognition accuracy than DL systems has risen dramatically. This is in line with a prior survey, which found that DL for tumor categorization, identification, and categorization has risen steadily since 2015 [91]. DL is being used in new ways to provide faster and more accurate assessments than behavioral scientists. The popular Google research for the analytic categorization of macular degeneration [106] revealed classification results far exceeding those of a healthcare professional. DL screening categorization must consistently perform under several situations, and the expected classification algorithm must be subjected to interpretation. For diagnostic categorization and diagnosis forecasting using DL to be ready for real-world therapeutic trials, as shown below in [52], many problems still need to be solved.

Professional intervention in pre-processing procedures for attribute mining and selection from imagery may be required in conventional ML methodologies. Nevertheless, because DL does not involve a social contact but rather extracts attributes associated with the input imagery, information pre-processing is not always required, resulting in greater adaptability in attribute retrieval related to different content-driven inputs. As a result, DL can provide a solid, validated version at any specified moment during the operation. Due to its versatility, DL has been demonstrated to work more effectively and efficiently than typical ML that depends on preprocessing [55].

Unfortunately, this component of DL inherently introduces ambiguity as to which aspects will be retrieved at each iteration, and so it is tough to describe which specific attributes were retrieved from the systems unless there is a dedicated architecture for the attribute [61]. It is also hard to ascertain just how these chosen characteristics contribute to a judgment and the comparative relevance of various attributes or subcategories of characteristics owing to the complexity of the DL algorithm, which includes several hidden units. This is a significant restriction for biological research, in which it is essential to comprehend the information quality of certain traits to create models. Ambiguities and inconsistencies risk obscuring the process of improving accuracy and making the correction of any prejudices that may exist in a specific content set more difficult. The application of the research findings to specific applications is also limited owing to a lack of transparency.

The problem of accessibility is related to the lucidity of ML outcomes but is not unique to DL. Due to its flexible concept, ML’s sophistication has made it easier to formally characterize. It becomes much more complex to describe why a certain forecast was generated as a perceptron develops into a neural net by integrating more hidden units. The categorization of AD using DL and three-dimensional integrative clinical information involves quasi-convolutional layers as well as the accumulation of distinct source dimensionality information, making it hard to perceive the influential factors of features extracted inside the actual data space. This forms obstacles with regard to the significance of the structure in the understanding of therapeutic images such as the “MRI”/“PET” scans. Additional sophisticated procedures create credible findings, but the scientific environment is hard to describe, though the outcome for analytical categorization must be transparent and comprehensible.

DL performance is affected by the pseudo random created at the beginning of training, and professionals can adjust hyper-parameters including “learning rates”, “batch sizes”, “weight decay”, velocity, as well as dropout ratios [111]. It is crucial to provide the same arbitrary values on numerous levels to obtain similar experimental outcomes. However, if the hyper-parameters and randomized samples are not supplied in most situations, it is vital to keep nearly identical software components [112]. The randomization of the training technique and the ambiguity of the setup could make it impossible to replicate the research and acquire similar findings. Whenever the availability of a neuroscience dataset is restricted, significant design consideration is required to minimize overfitting and consistency concerns.

In ML, security breaches happen whenever the information set architecture is structured poorly, leading to a system that utilizes unnecessary extra details for categorization [113]. Any successive MRI scans must be categorized as relating to a person with AD in the event of clinical categorization for neurodegenerative AD. When a participant’s neural substrates are covered by both training and validation, the structure of the brain region, not cognitive indicators, has a major effect on how they classify things.

Further studies should examine major discoveries using DL on completely different information sources. This is now generally known in genomics [114] as well as other domains, although DL research using neuroscience information has been slow to catch on. Furthermore, the growing open ecosystem of clinical study results, particularly in the domain of AD and associated symptoms, will provide a foundation for addressing this issue.

## 7. Conclusions and Future Work

DL methods and applications are constantly improving, leading to an improvement in the results in restricted scenarios such as picture identification. Whenever deduction is legitimate, i.e., whenever the training and test settings are identical, this helps to effective communication. This is particularly the case when employing neuroimaging to examine AD. Whenever the channel’s sophistication is too high to ensure openness and repeatability, one of DL’s flaws is the complexity of adjusting for probable network bias. This problem could be solved by collecting vast numbers of brain images and examining the correlations between DL and other attributes. The problem of consistency could be fixed if the variables used to obtain results and average scores from enough experiments were made public.

Deep learning is not a panacea for all situations. DL has problems adjusting to diverse types of information as a source, such as neuroscience with genomic information, because it retrieves properties associated with the input information minus the pre-processing for attribute choice. Since the weights for the input information are routinely adjusted inside a network, adding more data to the network produces ambiguity and uncertainty. On the other hand, a fusion technique separates the detailed data into ML components and the neuroimages into DL components while combining the two sets of results.

By solving these challenges and proposing problem-specific remedies, advancement in DL will be accomplished. DL techniques will become more effective as more information becomes available. The development of two-dimensional CNN into three-dimensional CNN is critical, particularly in the research of AD that involves heterogeneous neuroimaging. Furthermore, “Generative Adversarial Networks (GAN)” could be used to produce artificial healthcare information for augmentation. In addition, “reinforcement learning”, a type of learning that adjusts to modifications while making its own decisions depending on the environment, may have medical applications. The DL-based AD study is still in its early stages, with the goal of improving effectiveness and accessibility. As the amount of modalities neuroimaging information and computational power grows, investigation into using DL to diagnose AD is moving forward towards a model that includes only DL algorithms instead of a hybrid approach, but methodologies to incorporate wholly distinct templates of information in such a DL network must be established.

## Figures and Tables

**Table 1:** DL techniques for feature selection on neuroimaging data.

Reference	DL Technique	Accuracy
[69]	SAEsoftmax” regression layer	>86%
[70]	3D-CNN	>87%
[72]	SAE SoftMax” regression layer	>90%
[73]	SAE DNN	>84% for AD/CN classification >82% for MCI to AD classification
[74]	3D CNN	>92% for AD/CN classification >72% for MCI to AD conversion
[75]	VoxCNN ResNet	>79%
[77]	2D CNN	>85%
[78]	3D CNN	>75% for MCI to AD conversion
[80]	SAE 3D CNN	>90%
[81]	Ensemble of 2D CNN and RNN	>91%
[83]	3D CNN	>95% for AD/CN classification >84% for MCI to AD conversion

**Table 2:** CNN architectures for detection of AD and other disorders.

Reference	Illness	CNN Model	Accuracy
[19]	AD	“Inception + LeNet5”	96.85%
[76]	AD	VoxCNN + VoxResNet	80%
[103]	AD	“Inception V3”	92%
[104]	Breast Cancer	“Inception V3 + ResNet50”	85%
[105]	Skin Cancer	“Inception V3”	93.33%
[106]	Diabetic Retinopathy	“Inception V3”	90.3%

## Data Availability

Not applicable.
